# Novel missense *COL2A1* variant in a fetus with achondrogenesis type II

**DOI:** 10.1038/s41439-022-00218-5

**Published:** 2022-11-15

**Authors:** Yukari Kobayashi, Yuki Ito, Kosuke Taniguchi, Kana Harada, Michihiro Yamamura, Taisuke Sato, Ken Takahashi, Hiroshi Kawame, Kenichiro Hata, Osamu Samura, Aikou Okamoto

**Affiliations:** 1 Department of Obstetrics and Gynecology, The Jikei University School of Medicine, Minato Ku, Tokyo Japan; 2 Department of Maternal-Fetal Biology, National Research Institute for Child Health and Development, Setagaya ku, Tokyo Japan; 3 Department of Clinical Genetics, The Jikei University School of Medicine, Minato Ku, Tokyo Japan

**Keywords:** Rare variants, Genetic testing

## Abstract

Achondrogenesis type II (ACG2) is a lethal skeletal disorder caused by pathogenic variants in *COL2A1*. We present a fetus with cystic hygroma and severe shortening of the limbs at 14 weeks of gestation. We performed postnatal genetic analysis of the parents and fetus to diagnose the disease. A novel missense variant of *COL2A1* [NM_001844.5: c.2987G>A, (p. Gly996Asp)] was identified, which led to the ACG2 diagnosis.

*COL2A1* is located on chromosome 12q13.11, encodes the alpha 1 chain of procollagen type 2, and is expressed primarily in cartilage^1^. Abnormalities in *COL2A1* can cause several heritable diseases, such as achondrogenesis type II (ACG2) (OMIM #200610), hypochondrogenesis, platyspondylic dysplasia (the Torrance type), spondyloepiphyseal dysplasia congenita (SEDC), spondyloepimetaphyseal dysplasia (SEMD) (Strudwick type), Kniest dysplasia, Stickler syndrome, and spondyloperipheral dysplasia^1^. ACG2 is the most severe disease, and affected fetuses are stillborn or die immediately after birth.

There are approximately 461 skeletal disorders, of which more than 100 are identified *in utero*^2,3^. They are difficult to diagnose and show a low prenatal diagnostic rate (approximately 30–70%) in fetal ultrasonography^3^. In this study, we report a case of cystic hygroma and shortening of the limbs in a fetus. A novel heterozygous missense variant of *COL2A1* was identified in the fetus, confirming the ACG2 diagnosis.

A Japanese couple was referred to our hospital for a thorough examination at 14 weeks and 2 days of gestation after being diagnosed with a cystic hygroma in the fetus at 12 weeks. They were a non-consanguineous healthy couple with a 30-year-old female and a 29-year-old-male. They had no family history of skeletal disorder (Fig. 1). It was their first spontaneous conception. No deformation of the cranial bone due to ultrasound probe compression was observed. The thickness of the cystic hygroma was 10.9 mm (Fig. 2a). Thoracic hypoplasia was suggested. The femur and humerus lengths, which were 4.5 mm (−4.1 SD) and 5.0 mm (−4.2 SD), respectively, revealed marked shortening of the limbs (Fig. 2b). The fetus was considered to have a severe skeletal disorder, but a detailed diagnosis could not be made at the time. Chromosomal analysis by amniocentesis and another fetal ultrasonography was suggested, but the couple insisted on terminating the pregnancy. At 14 weeks and 6 days of gestation, an induced abortion was performed. The infant weighed 40 g, was 10.5 cm long, and had marked limb shortening and a cystic hygroma (Fig. 2c, d).

**Fig. 1:**
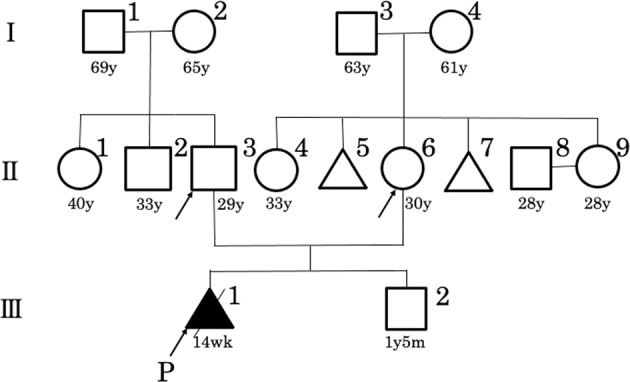
A family pedigree. A family pedigree of the Japanese family affected with achondrogenesis type II. P: proband.

**Fig. 2:**
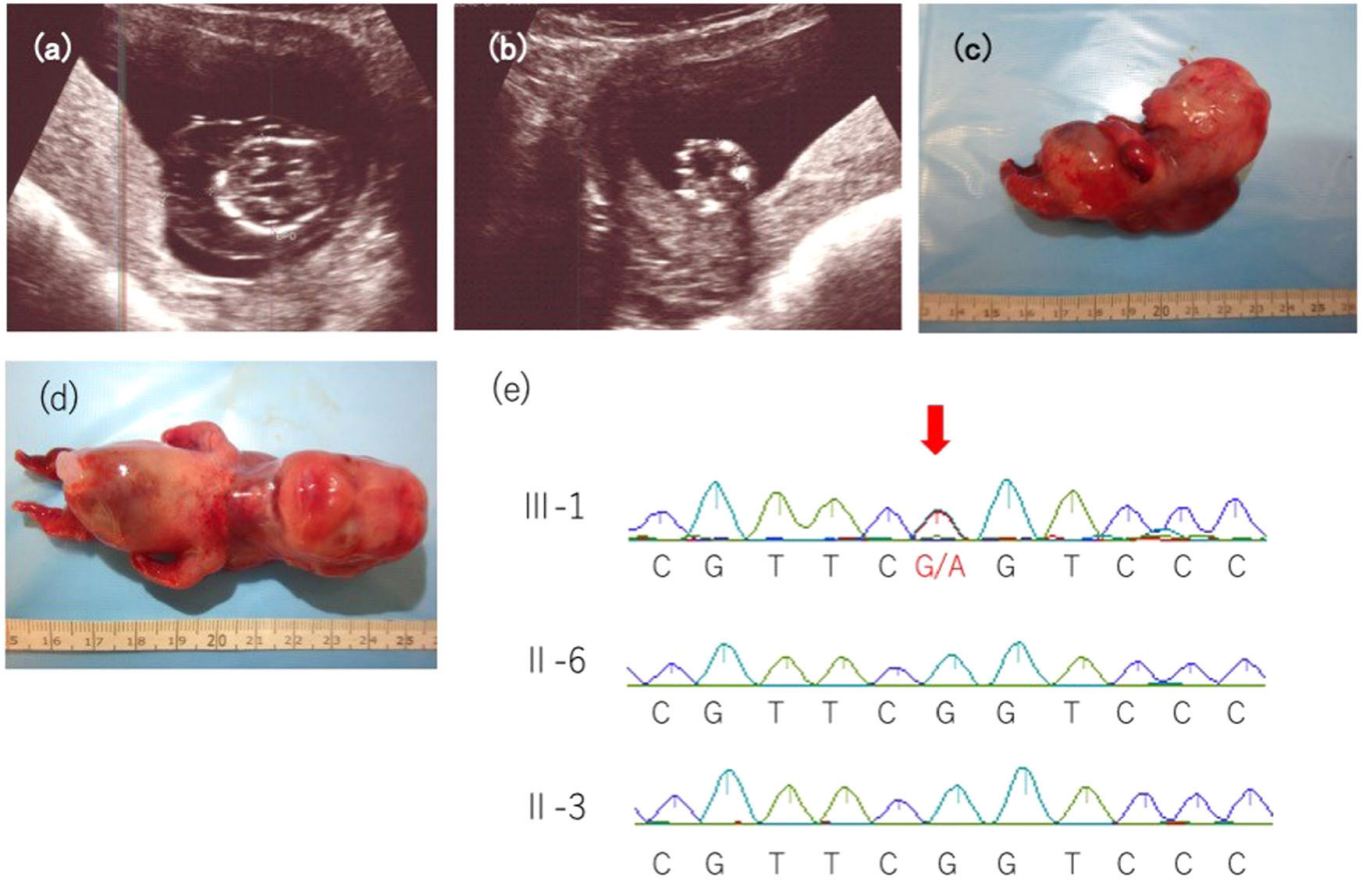
Fetal ultrasonography, clinical imaging, and genetic analysis. **a** Ultrasonography of the fetus at 14 weeks of gestation showing a cystic hygroma of 10.9 mm and **b** a femur length of 4.5 mm (−4.1 SD), depicting marked shortening. **c**, **d** Clinical image of the affected fetus. The fetus had markedly shortened limbs and a cystic hygroma. **e** DNA sequences of II-3, II-6, and III-1 to detect the *COL2A1* variant. The arrow indicates the novel variant that we detected in III-1.

Radiography taken after delivery showed marked shortening of the extremities. However, a detailed examination of ossification was difficult due to the fetus’s immaturity. Chromosomal analysis (G-banding) of the products of conception revealed a normal female karyotype (46,XX).

Following genetic counseling of the couple and obtaining written consent, we performed whole-exome sequencing (WES) for a disease diagnosis. The study was approved by the Institutional Review Board (IRB) of the National Center for Child Health and Development and the Jikei University School of Medicine [IRB number: 234 and IRB number: 27-060 (7945)].

DNA was extracted from the peripheral blood samples of the couple (designated II-3 and II-6 as per pedigree chart: Fig. 1) and the umbilical cord of the infant (III-1) using a previously described method^4^. A whole-exome library was prepared from the DNA samples of III-1 using the Agilent SureSelect v6 Capture Kit (Agilent Technologies, Santa Clara, CA, USA) following the manufacturer’s protocol. The libraries were sequenced on a HiSeq2500 (Illumina, San Diego, CA, USA) in the 101 bp paired-end mode. Sequence reads were mapped and aligned to the reference genome sequence hs37d5. Multisample calling of single nucleotide variations and short indels was performed with the RefSeq gene database in combination with 12 in-house control datasets. We extracted 1020 variants using our previously described method^4^. In addition, we extracted the variants with minor allele frequency (<0.01) according to the Integrated Japanese Genome Variation Database (ToMMo (iJGVD) 3.5KJPN; https://jmorp.megabank.tohoku.ac.jp) information. Finally, a novel heterozygous missense variant [NM_001844.5: c.2987G>A, (p. Gly996Asp)] of *COL2A1* was found to be consistent with the diseased phenotype. None of the remaining variants were associated with skeletal disorders. This variant was not reported in any control genome database, such as the International Genome Sample Resource (IGSR; https://www.internationalgenome.org), the Human Genetic Variation Database (https://www.hgvd.genome.med.kyoto-u.ac.jp) or ToMMo (iJGVD). This variant was not reported in the Human Gene Mutation Database (https://www.hgmd.cf.ac.uk/ac/index.php) or LOVD-COL2A1 (https://databases.lovd.nl/shared/genes/COL2A1). This change results in a predicted substitution of glycine 996 with asparagine acid (p.Gly996Asp). The SIFT and PolyPhen2 scores were 0 and 0.999, respectively, confirming the deleterious effect of the missense variant on protein function.

To confirm the variant found in WES and whether the couple carried the same variant, we performed Sanger sequencing of the DNA from III-1, II-3, and II-6 using a previously reported method^4^. The missense variant was observed only in the affected fetus (III-1) and not in the couple (II-3 and II-6) (Fig. 2e). According to the American College of Medical Genetics and Genomics guidelines, the variant was considered “likely pathogenic (PS2+PM2+PP3+PP4)” ^5^. The fetus was diagnosed with ACG2 resulting from a novel missense variant of *COL2A1*.

As mentioned above in the couple’s inspection, this was a possible de novo variant. After informed consent was obtained, the female had a second spontaneous pregnancy and gave birth to a healthy child (III-2) with no symptoms of skeletal disorders.

ACG2 is an autosomal dominant fatal congenital skeletal disorder caused by defects in the *COL2A1* gene^1^. *COL2A1* is located on chromosome 12 and encodes a polypeptide chain of type 2 collagen. The triple-helical domain, which is the backbone of type 2 collagen, consists of a glycine-X-Y repeating motif. Glycine substitution in this domain causes ACG2, hypochondrogenesis, platyspondylic dysplasia (the Torrance type), SEDC, and SEMD (the Strudwick type)^1,6^. In this case, a substitution of glycine for asparagine acid was observed (p.Gly996Asp).

Prenatal diagnosis of skeletal disorders can be performed by fetal ultrasonography or computed tomography (CT)^3^. For postnatal diagnosis, radiographic examination and genetic analysis can be considered. In this case, fetal CT was not performed because of the early gestational period. The specific symptoms of ACG2 include fetal edema, marked limb shortening, a bell-shaped thorax, nonossification of the vertebral and pelvic bones, normal skull ossification, and internal rotation of the toes^1,3^. In our study, we observed a cystic hygroma and marked limb shortening in the fetus. There were some reports of cystic hygroma in ACG2, and the findings, in this case, were considered consistent with ACG2 ^7,8^. Since the fetus was immature, ossification could not be evaluated^3,9^. Therefore, we performed WES to differentiate ACG2 from other skeletal disorders and found a novel missense variant of *COL2A1*. Based on the clinical findings and genetic analysis, we diagnosed the fetus with ACG2.

Somatic and germline mosaicism is also found in some parents of ACG2 patients^7,10,11^, which when present at low levels, are difficult to detect by Sanger sequencing using peripheral blood DNA samples. Therefore, if parents are suspected of having somatic or germline mosaicism, based on the family history, a close examination should be considered^7,11^.

## HGV database

The relevant data from this Data Report are hosted at the Human Genome Variation Database at 10.6084/m9.figshare.hgv.3246.
